# (Self)care by Numbers: Self-Monitoring Technology and the Technology of a UK Public Health Trial

**DOI:** 10.1080/01459740.2025.2471920

**Published:** 2025-02-28

**Authors:** Rebecca Lynch

**Affiliations:** Department of Social and Political Sciences, Philosophy and Anthropology University of Exeter, Exeter, UK

**Keywords:** Care, public health, research trial, self-monitoring, technology, UK

## Abstract

In an era of increasing interest in self-monitoring technologies to improve population health, this article considers how participants in a public health trial engaged with such technologies. Exploring how their engagement sits with the logic of self-monitoring and the technology of the trial highlights that the trial’s blackboxing of its objects of study obscure the deeply contextualized care practices through which such technologies “work.” Attending to (self-)care and what the trial neglects offers a means of disrupting entrenched values in its objects, relations, and logics, questioning what is important and for whom through a critical anthropology of/through health technology.

## MEDIA TEASER

I explore self-monitoring technologies and how these sit within trial technologies, attending to (self-)care and other neglected things as a potential means of disrupting biomedical values.

In an era of increasing interest and investment in self-monitoring technologies and their potential for improving population health through individual reflection and willpower, this article considers the use of such a device within a public health trial. I explore not only how trial participants engaged with this technology (explicitly designed on principles from health psychology) but how this sits with the logic of self-monitoring and the wider technology of the trial. Participant accounts suggest that self-monitoring practices here are better conceived of as care practices. This approach also highlights that the objects of the trial are more intertwined, relational, and dynamic and less separate and discrete than the trial presents these to be. While the trial objects are blackboxed as separate from context in order to assess effectiveness, instead participants’ accounts suggest it is through deeply contextualized care practices that such technologies “work.”

Drawing on Annemarie Mol’s logics of “choice” and “care” (2008), and more widely on work around the ethics of care, this article aims to contribute to a critical anthropology of, or indeed through, (health) technology, not only by exploring self-monitoring devices but the technology of the trial itself and the relationship between the two. In doing so, I follow Puig de la Bellacasa’s (2011) ethico-political approach in attending to care and other neglected things as a means to disrupt or intervene in the way through which particular objects, relations, or logics are understood.

The United Kingdom (UK) and other European countries are increasingly investing in self-monitoring technologies as one of the most efficient and cost-effective^1^ means of providing health care and health promotion (European Commission 2015; NHS 2019). Advocates of these technologies within biomedicine, government, and the technology sector stress their potential to prevent and reduce the burden of lifestyle diseases (Ruckenstein and Schüll 2017), self-monitoring technologies “empowering” people to change their lifestyles (Pols et al. 2019; Schwennesen 2017). As well as holding promise for clinical care, self-monitoring has been embraced by public health as a potential population health tool (Pols et al. 2019).

There has been a swift growth in the number of tracking devices and phone apps that sense health and movement over the last 10 years (Schwennesen 2017). These record and categorize bodily data that would otherwise be difficult to obtain (Ruckenstein 2014). Like technologies that have been around for many years such as thermometers, weighing scales, and blood pressure monitors, tracking devices are usually purchased independently from health-care settings and can be utilized at the user’s discretion. Self-monitoring technology itself is not “new” therefore. However, novel devices and their data have particular appeal and promise, and through the growth of groups such as the Quantified Self movement, recording, and sharing bodily activity, measurements are taken to an arguably almost obsessive extreme (Dudhawala 2018). Public health trials (such as the trial considered here) instead aim to discover how these technologies are used by the general population in relation to broader health goals, rather than by those enamored with self-measurement technologies.

The imperative to improve population health in what is termed the “new” public health (as opposed to earlier sanitary and infectious-disease focused public health approaches (Armstrong 1993; Dew 2012)) is frequently undertaken through a focus on an individual’s particular “health behavior” which are viewed as separate, discreet, and measurable (rather than on wider social structures, for example).^2^ In the UK university public health research unit where I undertook this work, there was a strong focus on what was termed “the four health behaviors”; smoking, diet, alcohol consumption, and physical activity, and the unit ran a number of different public health trials that aimed to find ways to change such behaviors. The discreetness and measurability of these behaviors as understood through this framing made them possible to include within a public health randomized control trial (RCT) (Adams 2016), where they can be linked to biomarkers indicating health improvement.

In public health more broadly, health behaviors are located within the individual and largely viewed as psychological processes (Cohn 2014). Self-monitoring technologies are seen as offering highly promising tools through which by changing individual health behaviors we might shift the wider bell curve of population health (Rose 1985). Individuals are seen to want to set, and then live up to, their own goals around healthy living, and are enabled by self-monitoring technologies to achieve this (Pols et al. 2019). The value of such devices is that in making people’s everyday movements visible, greater control over the body is possible, making it more amenable to change (Ruckenstein 2014). As such, ethics and psychology come together in health promotion discourses, with the value of tracking technologies being based on the idea of “a free subject that needs support to enact its will” (Pols et al. 2019:98). Within the trial, changing health behavior (improved levels of physical activity) is the goal (the object to be intervened on), with measurement of particular biomarkers as a proxy for this. The self-monitoring technology is the intervention seen as creating this change through “empowering”^3^participants by giving feedback on their behavior enabling reflection and health-promoting decision-making about future behavior. As such, the trial created three key objects: the body (measurable through biomarkers), the technology, and the rational, reflective psychological processes of the individual.

Seen in this way, self-monitoring technologies and their role in health promotion can be compared to Mol’s (2008) framing of a “logic of choice,” through which a focus on rational choice ignores the wider range of practices and discourses present in the “doing” of care. Technologies are viewed within a logic of choice as tools, or “means,” to reach particular “ends” (2008:57). However, technologies act in unexpected ways, and with unexpected effects, and it’s here Mol’s other logic, that of “care” creeps in. The trial seeks to document the effectiveness of the technology on the basis of psychological assumptions about rational reflection embedded in the design and expected use of the devices. However, through accounts of trial participants, the device becomes unhooked from the logic in its design and part of wider dynamic caring relations and interactions rather than driven by individual rational reflection. Indeed, while excluded within the framing of the trial, participants’ crafting and different forms of engaging in self-monitoring practices within everyday living are key to how these technologies “work.”

## Care, technologies, trials

The proliferation of “self-monitoring” or “self-tracking” devices has led to a proliferation of studies on these technologies, including those used for monitoring chronic conditions (e.g., Pols et al. 2019; Weiner and Will 2018; Will et al. 2020), preventative health (e.g., Oxlund 2012; Pols et al. 2019; Ruckenstein 2014), rehabilitation, fitness, or improved sports performance (e.g., Kristensen et al. 2021; Mopas and Huybregts 2020; Schwennesen 2017) or as part of the Quantified Self movement (e.g., Kristensen and Ruckenstein 2018; Kristensen and Prigge 2018; Dudhawala 2018). Many of the studies cited here take an approach to self-monitoring devices that explores them in relation to ideas of care, through which bodies and technologies appear as situated, interconnected, and relational, and part of wider socio-material care and everyday practices. Such approaches rub up against more limited framings of self-monitoring as surveillance technology. Placing the self as a responsible agent, it is easy to situate digital self-monitoring as another form of neoliberalism and means of control (Lupton 1997), and many authors have noted the (over)attention paid to surveillance and Foucauldian discipline and biopower in this field (Oxlund 2012; Pols et al. 2019; Ruckenstein 2014; Schwennesen 2017; Will et al. 2020).

Since the late 2000s work on care has burgeoned in anthropology and related disciplines (Buch 2015). Often drawing on the work of political theorist Joan Tronto (1993) and other feminist ethics of care scholars, this lens has moved activities often seen as unimportant and situated within the private sphere into public view (Mol 2008; Mol et al. 2010). For Puig de la Bellacasa (2011, 2017), this is an ethical move, giving “ethico-political significance” (2011:94) to practices that may be neglected or ignored and helping to disrupt entrenched values and to question what is important, for whom, and when. Through the dominant framing of the trial, self-monitoring technologies operate as an internal psychological process, separate to the body or the technology itself. Once reconfigured through the lens of care, however, different aspects of these interactions come to the fore. Care is not oppositional to technology and moves us away from viewing technologies as rationalist devices to part of embodied practices (Mol et al. 2010).

While notions of care may seem ill-fitting in the context of a public health intervention RCT (the technology of the trial itself not being amenable to recognize or measure such practices), accounts from trial participants make evident how central and relevant this is despite its apparent exclusion. Furthermore, attending to aspects neglected by the trial highlights the instability of the trial objects themselves, including the limitations inherent to the framing of the psychological mechanism through which the trial intervention is understood to “work.” Anthropologists are sometimes given a role in relation to such trials for example, in gathering experiences of trial participants. There is a risk here, as in other anthropological work in similar fields, of slipping into accepting the framing of fieldwork objects from within biomedicine or public health and in interpreting the social world through these. A critical approach therefore includes not only considering what the trial itself does as a technology (e.g., Dumit and Sanabria’s 2022 understanding of the trial as a form of colonialism) but in exploring the objects of the trial itself and how these may hold or break down in practice. Attending to the difficult-to-measure care practices neglected by the trial may be to follow Puig de la Bellacasa in disrupting entrenched values and to question what is important, for whom, and when.

In re-casting self-monitoring in practice as (self-)care then, I aim to move beyond and critique the framings of these technologies and their use within the trial, “thickening” what is otherwise a surface-level model of the social world and person–technology interaction. I use “self-care” hesitantly here and sometimes interchangeably with “care,” as while focused particularly on actions to care for oneself, this is not undertaken by, or restricted to, an individual “self” separate from caring for/by others. Made up of a multitude of different caring actions across differing components, what might be seen as self-care here is relational, distributed, and processual, undertaken through continual crafting. The imposition of the trial objects as discrete entities in relation to participants’ practices fitted poorly within the thick relational dynamics and nuanced active and co-constitutive sociomaterial interactions taking place.

This article is based on research on the “Get Moving” study, a UK-based RCT run from a university public health research unit between 2012 and 2014. The trial aimed to improve levels of physical activity through the use of self-monitoring technologies (for further details of the trial see Cooper et al. 2015; Lynch and Cohn 2015).^4^The trial developed its own accelerometer to record activities and provide feedback to users and, as noted in the trial protocol (Cooper et al. 2015), was based on psychological theories of change and motivation (e.g., Ajzen 1991; Bandura 1977; Leventhal et al. 2003). These theories are commonly used in relation to behavior change interventions (Holman et al. 2018) and were not themselves examined by the trial, being taken-for-granted as well established. Through these psychological theories, recording, and monitoring physical activity provide feedback to the wearer, motivating individuals to increase their activity. This increase in activity would ultimately improve participants’ health (as measured through different biomarkers) between data collected at baseline and at follow-up.

Over the 18-month period during which the trial was conducted, I observed trial clinical measurement sessions (which participants attended at the start and end of their 12-week trial intervention period) and spent time with the trial researchers. I observed and participated in meetings, seminars, and interactions within the research unit, presented and discussed my research, and took part in e-mail exchanges and social gatherings, allowing me to become familiar with the logic behind the trial and areas of concern (of most importance) in conducting the trial. I interviewed 30 trial participants about their experiences of using the self-monitoring equipment in their everyday lives^5^ and draw on participants’ accounts to explore and critically engage with, how these sit against the objects of the trial. Through these accounts, psychological understandings of self-reflection and willpower are thrown into sharp relief, while the embedded relational and co-constitutive dynamics of care illustrate the complex interactions that the trial contributes to but struggles to capture. Firstly, however, I briefly explore the objects and technology of the trial, the concerns of the trialists, and the materiality and functioning of the devices themselves.

## Technologies in/of the trial

RCTs enable comparison, under controlled conditions, of two or more therapeutic interventions (one of which may be standard practice/treatment, a placebo, or no intervention), with statistical analysis of the possibility of error (Meldrum 2000). For statistical analysis to occur, objects need to be rendered as comparable and countable and in relation to public health trials of human behavior, this includes complex social practices (Adams 2016). This process of rendering measurable objects from complex social practices and interaction, of inclusion, exclusion, and categorization, is not an apolitical or neutral move (Adams 2016; Bowker and Star 2000). As well as delineating objects from their wider context, the process forefronts those elements considered to be most valuable or important, made visible through countable proxies. It focuses on presumed relationships between cause and effect, holding steady and separating the intervention/treatment being tested, the target of the intervention, and the logic or mechanism of the intervention, relegating other aspects to the background, or as aspects that need to be “controlled” for. This facilitates analysis of otherwise complex objects through the technology of the trial but is also designed to allow the “magic bullet” – the trial intervention – to clearly “shine” (Dumit and Sanabria 2022:296).

As such, the Get Moving trial can be seen to have three key objects necessary to facilitate comparison through the technology of the RCT: the self-monitoring intervention technology or “active treatment” (Meldrum 2000); bodily biomarkers (the target of the intervention); and the mechanism that causes the former to influence the latter (conscious, internal psychological reflection and decision-making). These are necessarily artificially separated and conceptualized as bounded entities to enable analysis, simplifying the relationship between these (enabling comparison of placebo or “control” and intervention groups) and distinguishing them from the messy environment in which they are situated to create “controlled conditions” (Meldrum 2000).

The control of the conditions of the trial was of great concern to the trial study team and was a key subject within trial project meetings as potential “confounders.” There was concern, for example, about potentially competing trials that participants might also take part in that may make it difficult to assess the effectiveness of the Get Moving trial, and concern about how to remove or reduce bias in baseline/follow-up measurements if the measurement team became “unblinded” as to the trial arm that the person they were measuring was in. It was also suggested that perhaps I should be blinded as to the trial arm that participants were in when I spoke to them, to reduce bias in my interviews.^6^This second issue speaks to a second key area of concern in the trial project meetings; the recording of the main outcome measure of the trial. These were clinical measures taken at baseline (at the start of the trial period) and follow-up (at the end of the 12-week trial period). From the trialists’ perspective, there was less known about, and less interest in, the precise activities that took place during the twelve-week participants were enrolled in the trial, a blackboxed period during which the self-monitoring was seen to be taken place. This was little discussed and what I had heard about this during my interviews was viewed with curiosity but was not seen as fundamentally altering the running of the trial or likely to impact its outcomes.

These divisions might be understood to operate as a necessary fiction required for a research technology more easily suited to testing the efficacy of discrete pharmacological or clinical interventions than the messier and less clearly defined work of changing health behavior.^7^But such divisions here are also based on a particular, culturally defined “psy” (Rose 1999) version of the self: the bounded psychological, reflective self, located within the rational mind. This is a version of self that can be trained into being (Furedi 2004; Rose 1999), and the self-monitoring device here not only draws on the “psy” notion of self but, if drawn on in the way imagined in the trial protocol, reinforces this version of self. This, of course, supposes not only that the technology is being used in this way but that this is the central aspect that influences behavior.

When the trial was first conceptualized, the activity monitors were cutting edge. Based on pedometer technology, they were developed as a collaboration between design engineers and psychologists. However, technology develops faster than the research process necessary for funding a university-based RCT. By the time the study had successfully been funded and obtained ethical approval, the monitors were already fairly out-of-date and clunky. Technologies such as Fitbits had emerged, and health apps on phones were widely available. Looking back on the trial, the technologies appear particularly outmoded and basic, though notably the psychological logic underpinning their use has not changed. When the trial activity monitors were first distributed, researchers told me, trial participants were often disappointed at the look of this “novel” technology and its functioning. This was evident too for many of the trial participants I spoke to, who thought the monitors “ugly” and “like police tags.” However, these small gray devices designed to be worn on the wrist and fastening with Velcro were viewed by both trialists and participants as key to participants’ ability to self-monitor the level of physical activity they undertook.

As I discovered in wearing and experimenting with the monitors, the use of this technology was far from straightforward, and rather frustrating. The device had to be charged through connection to a power supply and then connected to computer software that first needed to be installed to function and for any data to be collected or displayed as there was no display panel on the device. The presentation of levels of activity on the computer screen was also clunky and presented broadly in graphs, making identification of particular activities or timing of these difficult. The technological feedback process was reliant on successful completion of these initial activities then and an ongoing process of charging and uploading. Furthermore, repeated use during the trial meant that many of the devices had ceased to function (something that was not evident until the charging, installing, and connecting process had been undertaken). The Velcro clasp and physical attributes of the monitors (a gray rubbery plastic box on a gray rubbery strap) meant that they could feel strange and slightly uncomfortable, with a tendency to come loose, and I, like some of the trial participants, lost the monitor when the Velcro failed. The actual use of the accelerometer was more complicated than the simple wearable device I heard described in the research meetings, and in the trial protocols and documents therefore. Again, discussions about technology issues reported by participants in the research office of the trialists, made very minor, if any, appearances in meetings. It was the environment, the “noise” around the trial objects that needed to be dealt with (or silenced) in order to allow the objective collection and processing of data of clinical biomarkers.

## The continual crafting of self-monitoring

While within the trial participants actively engaged with the feedback created by the monitors, this was not as the bounded, reflective, rational agents as conceptualized by the psychological models underpinning the trial. They also engaged with the activity monitors themselves as part of wider caring relationships: such technologies not only took a caring role but needed to be cared for. These three kinds of engagement – or forms of engaging, since they were ongoing – are focused on in turn in the following sections and illustrate the continual crafting required to undertake self-monitoring. Self-monitoring practices had to be undertaken within and around other everyday practices, sometimes fusing with, stimulating, or hitting up against these, and they were difficult to hold as stable and separate internal psychological processes. Instead, participants, technologies, and the wider circumstances in which these were situated actively co-created self-monitoring through dynamic, caring relationships and continual crafting; what I view as self-care.

### Care by numbers: Embodying metrics

Markers are useful to conceptualize and make sense of the abstract concept of “health,” and trial participants described using a range of different kinds of markers in relation to this. Many talked about noticing when and how easily they felt out of breath (for example, when they went up the stairs) and compared themselves to others undertaking similar activities (for example, how fast and far others could run, or how easily they lifted particular weights). Health was explicitly related to body weight by some participants, with a number describing how they noted they had put on weight through their clothes being tighter or needing to use another notch on their belt. Bodily interaction with the material world as well as physical bodily experiences were ways through which abstract ideas of health were grounded and linked to everyday life and one’s own body. Before using any technologies, then, participants already engaged in somewhat lower-tech and rougher versions of self-monitoring.

Through such measurements, the body, and health, can become objectified, with health taken out of being a corporeal experience and made into a numerical object. This is no longer about how one feels, or a rough comparison to others, but it comes through a precise “objective” measure, independent of less reliable subjective readings, a “datafication of health” (Ruckenstein and Schüll 2017). This was a helpful upgrade for trialists who no longer needed to rely on people’s own assessments (which were understood as unreliable “self-report” data), and like other measurements, such as cholesterol and blood pressure, these technologies provided individuals with data points to compare with others and wider norms. Once monitors were plugged in and their data uploaded to a computer, participants could see how active they had been on a particular day and over a week (depicted as a visual illustration on the screen) and were provided with another numerical means through which health could be understood.

The numerical data these technologies generate are situated in relation to particular bodies; one’s own numbers. As such, health is reinforced as an individual attribute within an individual’s body, created through individual health behavior. The rationale of health feedback technologies is that these numbers are then reincorporated as an experience; the numbers come to be, or replace, subjective health markers such as how one feels. This more reliable objectification of health can be internalized by the individual and this becomes embodied; I am active because I have a high-step count. However, this is a particular kind of embodiment. It is not a neutral process but is guided by public health psychological understandings that these particular numbers are important and should result in action; my physical activity (my step-count) should increase so I improve my health. As such, these technologies, and the numbers they produce are based on particular choices and values, reinforcing these and a public health emphasis on individual responsibility for health; these are my numbers and therefore are my responsibility.

Such numbers inevitably emerge from measuring what is possible to be measured; what can be quantified, and the tools created to record it. For example, recording activity became easier with the invention of the electronic pedometer (Bassett and Strath 2002), which measured body movements it calculated as “steps.” “Steps” then became an indicator of levels of activity. Activity levels might also be measured through heart monitors, which look at increased or decreased heart rate as a proxy for movement. These different technologies – pedometers and heart monitors – both produce measurements of body activity but through different *types* of measurement that give *different sorts* of readings for interpretation. They are also worn and used differently. Heart monitors are expensive, need to be worn constantly and are fixed by clinical professionals on to specific sites on the chest. Data produced by these needs to be uploaded within a clinic. Pedometers can be easily taken on and off by users themselves, are easily readable through a computer program or visual display and are cheaper to produce. The pedometer is therefore far easier to employ as part of a behavior change RCT looking at self-monitoring and is more likely to be or become commercially available. However, this does not give a “better” reading of activity.

The Get Moving trial used a more advanced form of the pedometer – an accelerometer – that measures rates of bodily movement in a more sophisticated way. It is designed to be worn on the wrist and gives an indication of activity in minutes classified as “very high,” “high,” or “moderate.” Heart monitors were also used, but only as part of the clinical examinations conducted at the baseline and follow-up time points. Two different monitors recording two different representations or versions of activity were therefore used within the trial. As such, therefore, the ability to measure something specific (for example, steps, heart-rate, or minutes of movement) and make this a proxy (biomarker) for physical activity, resulted in particular kinds of readings coming to stand for health at different points. In this trial, the minutes and intensity of movement as measured by the activity monitor were the focus for participants so that increased movement indicated increased physical activity and became the gateway to improved health and fitness. However, the trialists themselves used and prioritized data from the heart monitors. These were considered “purer” and more objective than accelerometer readings, since heart monitors were placed on the body by trained clinical staff in the lab (at baseline and follow-up), and these were seen as giving a superior and more nuanced reading of activity suitable to draw conclusions from as part of the trial. Contrary to the expectations of trial participants, the trialists did not use the accelerometer data.

Self-monitoring technologies reify and reinforce particular understandings of health through particular kinds of data. For instance, an increased movement rate on my monitor equals improving my health, in turn reifying and shaping our expectations of what health is and what care for this might be. From the trialist’s perspective, this form of feedback, ensuing reflections, and the resulting changes to behavior by the individual form a simple psychological loop, something that might be understood as rational attending. This is a quantification of life suggesting that a calculable way of achieving a goal is possible. However, as Pols, Willems, and Aanestad note, “the perfect calculation can never be carried out” (2019:109), the future is not possible to predict so directly and data generated is never separate from its dynamic and changing circumstances and interpretation.

Trial participants sought to place numbers generated by their activity monitors into a wider context. As new and unfamiliar kinds of readings, what did such digits mean? Were they good or bad? How did they relate to other people’s numbers over a day or a week? Some interviewees talked about their frustration at not having “averages” they could compare themselves to. For example, Jane, a 22-year-old data clerk, talked about not only her desire to be near “the top of the chart” but to understand herself as “above average” in terms of her level of physical activity. She wanted to make monitoring a competition between herself and other women of her age. Other participants talked about experimenting with the readings produced by the activity monitors – what increased or made little difference to these? Many placed them within their wider subjective experience of their activities, for example, in walking to work or attending an active gym class. This was easier for some activities than for others. The numbers “made sense” when they gelled with people’s own experience, for example, a day when participants were aware of having been more active being picked up as such by the technology. Jane told me she went to her first kick-boxing class while wearing her activity monitor. She was impressed at the amount of activity picked up by the monitor, and this enthused her about these classes, and she started to undertake kickboxing as a regular activity. The numbers produced by the monitor appeared to Jane to match with the activity she was undertaking.

When participants felt they had been active and this had not been picked up, the effectiveness of the technology was often doubted. A number of participants told me they thought the monitor was limited by not recording cycling in the same way as it recorded walking, as the monitor was placed on the wrist (which was largely immobile during cycling) and not on the ankle (which was peddling). Some participants sought to remedy this by changing where they wore the monitor, moving it to their ankle or around on their arm. This altered position and use of the monitors came as a surprise to the trial research team when I discussed this with them. The idea that participants might wear activity monitors that were designed to be worn on the wrist (and calculating movement on the basis of this) anywhere else on the body was unimagined.

Evidently, not everyone who participated in the trial was engaging with the numbers generated to the same extent – some people told me they were not interested or could not get the technology to work for them. Others talked about setting targets and goals based on the numbers they were producing. For example, Chloe, a 49-year-old accountant, had targets she was aiming for, and she wished that the activity monitor computer program would note when she had reached them through displaying a reward icon in the same way as her Weight Watchers app did. She told me that she found these forms of recognition powerful; “I think those sort of things, although we’re all grown-ups, it is like getting a star when you’re at school, and it does give you a little bit of a ‘yay’ moment.” For Chloe, and for others in the trial, the numbers produced by the monitors were often combined with measurements of weight or other markers, such as clothes fitting better or their bodies appearing “more firm.” Even with these monitors then, numbers were not the only marker participants relied on; subjective experiences slipped in and were not subjugated by quantitative readings (Mopas and Huybregts 2020). Numbers and targets were compared to and discussed with other people, and targets were sometimes set to be achieved with colleagues or with friends and family. Self-monitoring was not always an individual activity, therefore, but part of social relationships and group targets, and as such, part of other caring arrangements (Kristensen et al. 2021; Weiner and Will 2018).

As Byron Good notes, numbers are a “symbolic form”; a “symbolically mediated mode of apprehending and acting on the world, by which medicine formulates sickness from a materialist and individualizing perspective” (Good 1994:197). Sickness is understood through numbers in biomedicine, and caring takes place through attending to these. Numbers retain a symbolic form, not only for clinicians but also for participants, becoming meaningful parts of lives, a “numeral ontology” (Oxlund 2012). Participants gave value to and made meaning through such numbers; they wanted to “beat” them, change them, share them, and situate them. They related to their numbers. As such, numbers could take on a somewhat “magical” quality, becoming a goal, a focus, or a kind of “quest” for participants. Numbers were productive, not only reductive, resulting in new meanings, practices, and socialities with lives of their own. While numbers and the technologies producing them might have created particular understandings of health and the body and emphasized a particular form of self-care, they were simultaneously situated within wider experiences and personal understandings. Self-monitoring of one’s numbers involved engaging, valuing, relating, situating, caring, all ongoing activities connected to the individuals’ circumstances.

It is perhaps worth noting that for trialists too, engaging with – and what we might also term “caring” for – the numbers being produced through the trial were essential. In meetings, over e-mail discussions, and through trial documents, those working on the trial and on other public health projects discussed and thought through the trial data in great detail. Datasets should be “clean” and missing data dealt with. Protocols were developed for collecting, handling, and storing such numbers. As mentioned above, there was great concern that the clinical staff involved in collecting objective measures from participants – such as drawing blood to give cholesterol readings, operating running machines measuring VO2 max (maximum oxygen uptake, a means of measuring aerobic endurance) or weighing and recording the waist size of participants – should be “blind” to which trial arm, a participant had been randomized to. This essential reduction of “bias,” or subjectivity, from creeping into an otherwise “objective” process (Mason 2018) through adhering to protocol was part of the work of separation of the active ingredient of the trial from the environment in which it took place.

### The self through feedback loops

The logic of the self-monitoring technologies employed as part of the trial was that trial participants would look at the numbers generated, reflect on these, change their behavior accordingly, and start the cycle of observing and reflecting again. Key to this observing/reflecting/acting was that this was an ongoing activity, a generative loop that participants repeated daily, or at least weekly. This loop itself created and reinforced, a conceptualization of “self” as independent, rational, and reflexive, and with the ability to change one’s own behavior. Improved health was possible through being cognizant of one’s behavior and the will and desire to alter this, the monitoring technology enabling this reflective process. Through the logic of self-monitoring, individuals planned and made decisions about what activity they would undertake in advance of undertaking the action, and this took place as a psychological process occurring within an individual’s head; the culturally specific, bounded and tight “psy” version of self, separate from body and environment.

Sociologist Chris Till (2014, 2018) has described the reflective process of self-monitoring as a form of labor. Not only is a particular and increasingly tighter version of self-being enacted but undertaking this can be cast as “work.” This position has resonance in the use of personal data by Facebook and Cambridge Analytica where individuals’ digital labor was used and sold by companies for their own benefit (Cadwalladr and Graham-Harrison 2018; Data workers of the world, unite 2018), but also connects to Protestant ideas of work as an end in itself through which other types of self-care emerge as a form of luxury. A focus on self has been linked to narcissism and “modern” living (Lasch 1991), as indulgent and somehow immoral. The cultural notion within the UK, or at least the conceptualization reinforced by marketing frequently aimed at a female market of “busy women” (especially mothers), presented as bearing an additional workload and range of caring responsibilities for others, also links to this idea, where “me time” is a “treat.” Idealized forms of self-care, such as holidays, spas, and beauty treatments, are presented as luxurious activities, reinforcing an opposition between “work” as a virtuous activity while these forms of self-care are “indulgent.” However, we might note there are other forms of self-care that are not necessarily recognized as such and are situated within the domain of virtuous “work.” Saving money to get on the property ladder or focusing on gaining a promotion, which might be argued to be important forms of self-care for the same group to whom such spa breaks are marketed, remain connected to “work,” perhaps through being more obviously situated within (capitalist) notions of responsibility, self-control, intellectual assessment, and planning. Requiring similar skills, self-monitoring is also well suited to being seen as a form of “work” – a type of moral labor virtuous to engage in, rather than an indulgence or luxury. To be an individual who self-monitors is to be “good” and “responsible” and particularly so in a context where health prevention is a moral imperative (Oxlund 2012).^8^

This process of feedback fits neatly into ideas of self-monitoring as a Foucauldian technology of the self or as a panoptical self-surveillance (Foucault 1988; Lupton 1997) and like self-monitoring-as-work, self-monitoring-as-a-technology of the self positions the individual as the driving locus of the loop. However, this self:technology relationship is more complicated. Through these technologies, the person learns to read their body based on numbers the activity monitor produces but in the resulting feedback loop the person is both subject *and* object (Pols et al. 2019). The technology gives feedback about activity, which impacts the activity, which impacts the feedback, which impacts the activity, and so on; I am the data, and the data is me. As Ingold (2014) notes of participant observation, we find a mess in researching and being researched, an entanglement of subject and object. Similarly, through this ongoing processual loop of feedback, there is an eventual erosion of these categories; the loops get tighter and smaller, more refined, predictable, and appropriate. Other things get left out; the activity data produces health, health produces activity data. The data-producing body is producing health. This undermines the fiction of an autonomous individual in these understandings that there is a rational reflexive self and a number-producing technology. At no point is there “the self” and “the technology”; neither self nor technology is autonomous, the loop is made up of both. Self becomes technology, technology becomes self: the bounded “psy” self is not so separate. Linking to Haraway’s (1991) cyborg breaking down human:technology dualisms, this is another version of self, which also excludes other versions as well as reinforcing itself. However, the emancipation from the boundaries of human and technology also allows understanding of feedback as care. The relationship is co-constructive, a form of caring that emerges from the relationship created through the feedback loop.

Take Wendy, for example, a 29-year-old personal assistant, who told me she wanted to participate in the trial as she had noted that her weight had crept up and she thought it would be a good way to be motivated to lose weight and to exercise. She had started to get backache, her clothes were getting tighter, and she said she wanted to “get back to health.” Through the activity monitor she was given as part of the trial Wendy noticed that she was not as active as she had previously thought she was. She told me she wore the activity monitor most of the time, it was not attractive to look at but otherwise did not cause her any problem to wear. It was like a watch, and she wore it all the time apart from showering.

Wendy looked at her activity readings once a week and also weighed herself. She described already knowing what the activity readings told her, but it “validated” what she knew she was already doing. Looking at her activity readings gave her a sense of achievement, and she wanted to be more activefor example, to climb the stairs rather than taking the lift. She walked at every opportunity and built this into her life taking longer routes to walk into work and then home. Wearing the monitor meant she knew she was being recorded and so she did more, and then got better results. While she described this as “Big Brother watching you” (a reference to a constantly surveilling leader in George Orwell’s dystopian novel *1984*, Orwell 2013 [1949]), she also talked about discussing the process with colleagues and was motivated to exercise with them also. As the weeks went on, she lost weight and started to feel better, feeling “more supple,” she said. Her back stopped aching and she saw herself as toning up. She went down a trouser size, and people noticed and commented on this. Wendy told me that encouragement from others and noticing her weight change was motivating her to continue wearing the monitor, and the monitor motivated her to do more walking. She could see progress each week with more activity, describing how activity led to fitness, which led to weight loss, which meant a healthier lifestyle.

At points in this description, it is not clear whether it is the activity monitor or Wendy doing the work here, both are part of an ongoing relationship that also brings in other concerns, forms of monitoring, bodily experiences, and social networks. At the time I spoke to Wendy, she had completed the trial and was continuing to walk and take the stairs and told me she enjoyed doing so. She did not need to look at the activity screen anymore, she said, but was keeping an eye on her weight, still self-monitoring, still attending to and taking care through this looping. Thinking toward her future she told me she wanted to be healthy in middle-age, the relationship between activity, weight, and health was continual and ongoing. Through such a loop she was caring for her future self as well as her current health.

### Technologies as care, caring for technology

The above form of self-monitoring delegates a caring role to technology. The activity monitors “stood in” for health professionals, taking up the role of producer and recorder of health data and therefore the monitor of a person’s health. This delegation was key to the design and enthusiasm of public health trialists about such devices; these technologies would become efficient forms of enacting care, not requiring appointment attendance or time from busy clinical schedules. Able to be scaled-up to populations and relatively affordable, such independent care could be provided on a large scale.

A considerable problem with this view of digital care is that such technologies are not available to all equally. For example, Fitbits are relatively expensive and are easier to engage with if you are already familiar with similar equipment. They suit some people and their lifestyles better than others. The datafication of health exists within a wider “digital divide” (Ruckenstein and Schüll 2017), and the potential for these technologies to increase rather than decrease health inequalities and levels of care should not be forgotten (Green 2018). In addition to critiques about who is ultimately able to gain from such technologies, there have been concerns these devices replace and exclude other forms of care (Pols 2012); through these technologies and the feedback loops they create, what counts is what can be counted (Adams 2016). However, rather than excluding other forms of care, the conceptualization of a sophisticated self:technology loop merely hides many more forms of caring that go on at the same time. The activities recorded by the monitors do not occur in isolation but are part of an individual’s everyday life and wearing a monitor fits more or less easily into these.

The delegation of care to these technologies was an aspect recognized by many participants who, like Wendy, talked of “Big Brother” watching their results. They sometimes described what they thought the trialists would be thinking as they looked at their daily activity data and talked about wanting to be thought of as a “good” trial participant. Participants were frequently disappointed that no one from the trial would be talking “their” data through with them, and often instead chose to discuss these with friends and colleagues. Many participants liked the idea of being watched or monitored in this way, and saw monitoring as part of care, whether by technology or others. Jane, for example, talked about preferring feedback from computers rather than from people, seeing these technologies as just “reporting facts and figures” rather than “judging you,” their “objective” readings an advantage. At the same time, Jane wanted to show her readings to others when she was doing well, the judgment of people frequently being more meaningful than the reports given by the monitoring technology. While these technologies provided care, then, valuing and appreciating the readings was seen as an important aspect that required other people.

Self-monitoring reinforced the caring relationships it was embedded in different ways. Time and space to be active as well as being with other people were key components of how trial participants gave accounts of their activity and self-monitoring, and self-monitoring could become a social activity with people becoming accountable to others as well as themselves. Participants described being motivated to go walking with friends with similar monitors and family members being involved in deciphering and attending to feedback readings. Some spoke to health professionals they saw outside the trial about participating in the study. As a workplace-based trial, many participants reflected on how they were not only now discussing what physical activity they did with colleagues but other aspects of their health more broadly; colleagues became involved in caring relationships in a way they had not been previously.

Wearing the activity monitor and generating and reflecting on the data received was only ever one part of what engaging with the intervention turned out to be. Activities undertaken by participants were part of everyday living and involved many more items beyond the accelerometer. For example, Sarah, a 57-year-old administrator, talked about using the monitor to measure her activity during playing tennis, an activity which did not only involve wearing the monitor but also included booking free tennis courts, packing racquet and balls, driving to the court, meeting her tennis partner, and wearing kit. The monitor was never the only form of technology involved in this bodily activity. It was also not the most crucial. Sarah explained how her weekly tennis playing had come to an end when her friend was no longer available to join her, and she therefore lost a major component in the social association of the self: technology loop.

The monitors themselves relied on being charged and on leads that connected them to computers, electricity, and plug sockets. As is evident in work on the anthropology of technology more broadly (e.g., Bruun and Wahlberg 2022), they were dependent on networks of other things and activities. While it was easy to wear the accelerometer, it was more difficult to download the data. Lindsey, a 47-year-old nurse described getting increasingly frustrated with the monitor. She described herself as a fairly active person but as someone who did not really exercise or weigh herself. She was “vegetarian with a very sweet tooth” but beyond this did not watch her food intake. Initially uploading her activity daily, and then more or less weekly, she had no regular routine, she would just get to “it” when she remembered. She disliked the presentation of results the computer gave, finding these cumbersome and crude. She was not able to distinguish how her readings related to particular incidents in her day and would sometimes forget to charge the monitor or download the data within the two-week time period the monitor stored information for.

These caring technologies required being cared for (Mol et al. 2010; Pols 2012). Some trial participants had to attend to the specifics of wearing the technology. As a nurse, Lindsey, for instance, was not able to wear anything on her wrists while at work so she placed the accelerometer higher up on her arm during this time. At one point in the trial, she was unable to wear the accelerometer for more than a week as she had not taken the charger on holiday with her. Many other interviewees also described attending to when, as well as where, the monitor was worn; not at weddings nor on bare arms in summer where it might look ugly or out of place, not swimming nor in the shower where it might get damaged. Trial participants attended to wearing the technology itself therefore, not only to the numbers it produced.

Caring for both humans and technologies requires more than one act of care, caring being made up of a multitude of different activities and contingent on other networks, technologies, and other forms of caring relationships. Care, even if considered “self-care,” is dependent on many others. While productive to view self-monitoring as a form of care, this should not be viewed as *the* form of care. To do so risks letting self-monitoring hide other caring forms or allows these to be discounted as there is no care at all. This importantly expands the understanding of self-monitoring from that suggested by public health behavioral approaches. Self-monitoring as a form of (self-)care can be seen as the quality of lots of different relationships and elements therefore, not just one.

## Conclusion

In noting the careful and creative crafting that trial participants were engaging in to undertake self-monitoring, the conceptualization of this as a form of care becomes more relevant being embedded within, and emerging from, the particular contexts of people’s everyday lives. What was meant to be (from the perspective of the trial) a very standardized form of self-monitoring, for participants was highly varied and personalized. For participants, self-monitoring involved different ways and degrees of engaging and “tinkering” (Mol 2008) with equipment, so that these technologies and their use fitted people’s everyday circumstances. Participants altered when and how they engaged in self-monitoring. This form of care like others (Mol et al. 2010) involved an ongoing relational process, a small and modest set of projects that participants tweaked over time to suit different circumstances.^9^This could be a social, not a solo, endeavor; there was not a singular individualistic “self” involved in self-monitoring (Lynch and Cohn 2015). As such, and in opposition to the framing of the trialists, we might suggest that there were as many interventions in the trial as there were participants. While on one level reinforcing a culturally specific and “psy” notion of self as separate, rational and reflective, this broke down in practice with self and technology looped together.

Trial participants engaging with numbers, feedback, and the accelerometer technology might appear to more-or-less “fit” with public health framings of self-monitoring and trial conceptualization of these as three separate elements: an intervention technology, body to be intervened on, and psychological process as an active ingredient between these. However, these elements were more entangled, dynamic, and expansive. Self-monitoring was a relational process, not tied solely to the technology or a loop created by participant recording and reflection but, as others have noted of care practices elsewhere, emerging from heterogeneous sociomaterial relations that come together to make and unmake each other over time (Mol 2008; Mol et al. 2010). It is therefore difficult to objectify care; to count it, measure it, and to detail precisely what it involves for each different person. Like proxy measures for health, attempts to chart care are likely only ever to be partial, caring occurring at a very local level and in many diverse ways. Care resists inclusion in an RCT, it does not make for a stable, measurable, clearly identifiable, comparable, scale-up-able research object or intervention (Adams 2016).

Understandings of self-monitoring as a universal psychological process are undermined through notions of care, hitting up against the guiding notion that individuals are rational, goal-achievers through assertion of their will (Pols et al. 2019). Self-monitoring instead emerges as an embodied, situated, and physically engaged set of practices, the relational aspects of this form of care challenging the value placed on being self-sufficient and independent from others (Puig de la Bellacasa 2011). Care as a concept takes us to the heart of ideas of the relationality of bodies and health (Mol 2008) running contra to the notion that we are the autonomous beings suggested by biomedicine. The body is not a given waiting to be observed but is in ongoing dynamic interplay with technologies and its wider surroundings (e.g., Mol 2002; Mol and Law 2004). A focus on accounts of public health self-monitoring devices within a trial illustrates the limits of the psychological assumptions and values embedded in their design, but also makes these ideas, and their connection to wider understandings and existing frames in biomedicine more obvious. People and technologies are positioned in specific relation to each other while care is left out of how these technologies are seen to work. Care is consequently overlooked, so that a continual focus on self-monitoring devices as psychological tools may, as Puig dela Bellacasa says of other sociotechnical assemblages “… reinforce asymmetrical relations that devalue caring” (2011:94).

Through conceptualizing self-monitoring as a form of care, we see how this is distributed across different social and material aspects and connections, drawing on particular values and understandings of the body and health, and calling us to attend to particular relationships. In fact, it is difficult to pin down exactly where and when care (self-care or otherwise) takes place in these different interactions and practices; where *is* self-care located? I argue that it is precisely through the particular forms of crafting and engaging that participants undertook that caring was “done.” Self-care *is* the crafting and connecting of these elements, their bringing together. Care happens across all of these forms of engaging and is not embedded within the device itself.

## Notes

1. The cost-effectiveness of these technologies in the eyes of biomedicine is connected to ideas of “empowerment” for patients, as patients are seen to take on a more active role in their health (Schwennesen 2017).

2. Not all public health focuses on this field, also being concerned with environments and infection, for example.

3. As well as the risk of empowerment becoming an “obligation” for the patient (Ruckenstein and Schüll 2017), this notion suggests that patients are passive in the receipt of care, ignoring the ways in which they are already actively involved e.g. in taking medicine, visiting their doctor etc (Mol 2008; Mol et al. 2010).

4. Participants were recruited from a large workplace which had many different types of jobs. Participants were “healthy,” having different levels of health but able to take part in the walking tasks undertaken during baseline and follow-up clinical measurements. Some participated because they specifically wanted to get healthier or lose weight, others felt motivated to join to “help” research and scientific progress, for others, it was the appeal of receiving cholesterol readings or the results of other clinical measures that would tell them about their health. Often, it was for a mix of these reasons.

5. Twenty-four of the participants interviewed were women and six were men, which roughly reflected the make-up of participants in the trial more broadly. Those interviewed were of a range of ages, 10 participants being between 20–34 years old, six between 35–49 years old, and the remaining 14 between 50–64 years old.

6. This was clearly not a feasible suggestion, nor desirable, as I needed to speak to people who had the intervention to ask about it, I would hear during the interview which trial arm they were in anyway, plus of course, concerns that this would “bias” my interviews in a problematic ways emerges from positivist understanding of qualitative research approaches.

7. Although of course drug and clinical trial are also themselves messier than is immediately obvious (Dumit and Sanabria 2022)

8. This is also interesting to consider in relation to how “care” itself is valued. Mol et al. (2010) note that terming “care” as “work” (such as situating care as a mother as a form of “work”) has been a way of adding value to practices around care.

9. As noted by other authors (e.g. Mol et al. 2010; Puig de la Bellacasa 2011), there is not one way to care but specific ways of caring for each situation.
